# The plus score as a dual-domain predictor of delayed cerebral ischemia and mortality after aneurysmal subarachnoid hemorrhage

**DOI:** 10.1007/s10143-025-04086-9

**Published:** 2026-02-07

**Authors:** Mehmet Sabri Gurbuz, Ece Uysal, Yunus Emre Ozbilgi, Deniz Alyanak, Simge Sezgin, Abdullah Talha Simsek, Burak Bayraktar, Hidayet Safak Cine

**Affiliations:** 1 Department of Neurosurgery, Istanbul Medeniyet University, Suleyman Yalcin City Hospital, Prof. Dr, Istanbul, Turkey; 2 Department of Neurosurgery, Acibadem Healthcare Group, Acibadem Altunizade Hospital, Istanbul, Turkey

**Keywords:** Subarachnoid hemorrhage, Intracranial aneurysm, World federation of neurosurgical societies grade, Modified fisher scale, Microsurgical clipping, Vasospasm, Delayed cerebral ischemia, Mortality

## Abstract

Delayed cerebral ischemia(DCI) is a major contributor to poor outcomes after aneurysmal subarachnoid hemorrhage(SAH), primarily due to symptomatic vasospasm. Accurate early risk stratification is essential for optimizing monitoring and treatment. Two commonly used predictors are the World Federation of Neurosurgical Societies(WFNS) clinical grade and the modified Fisher radiographic grade. We assessed whether the simple arithmetic sum of these two scores—termed the “Plus score”—could improve the prediction of DCI and mortality. We retrospectively reviewed the consecutive patients treated with surgical clipping at a single tertiary center from 2021 to 2025. Inclusion required angiographically confirmed aneurysmal hemorrhage, clipping within 72 h, and ≥ 6 months of follow-up. DCI was defined as a new neurological deterioration between post-SAH day 3–15, excluding the other causes. Logistic regression and receiver operating characteristic(ROC) analysis were used to compare the predictive performance of the WFNS grade, modified Fisher grade, and Plus score. Fifty-nine patients (mean age 53 ± 14 years; 52% female) were included. DCI occurred in 32 patients(54%), and in-hospital mortality was 16.9%. The mean Plus score was significantly higher in patients with DCI than those without, and higher in non-survivors than survivors. The Plus score showed superior predictive value for DCI (AUC = 0.748; cutoff ≥ 4.5: sensitivity = 84.4%, specificity = 55.2%) compared to WFNS (AUC = 0.704) and modified Fisher (AUC = 0.703) scores. For mortality prediction, the Plus score had an AUC of 0.870. The Plus score, combining neurological and radiographic severity into a single value, offers improved risk prediction for DCI and moderate accuracy for mortality. Its simplicity and clinical accessibility make it a valuable bedside tool for early stratification and targeted care in patients with aneurysmal subarachnoid hemorrhage.

**Graphical Abstract:**

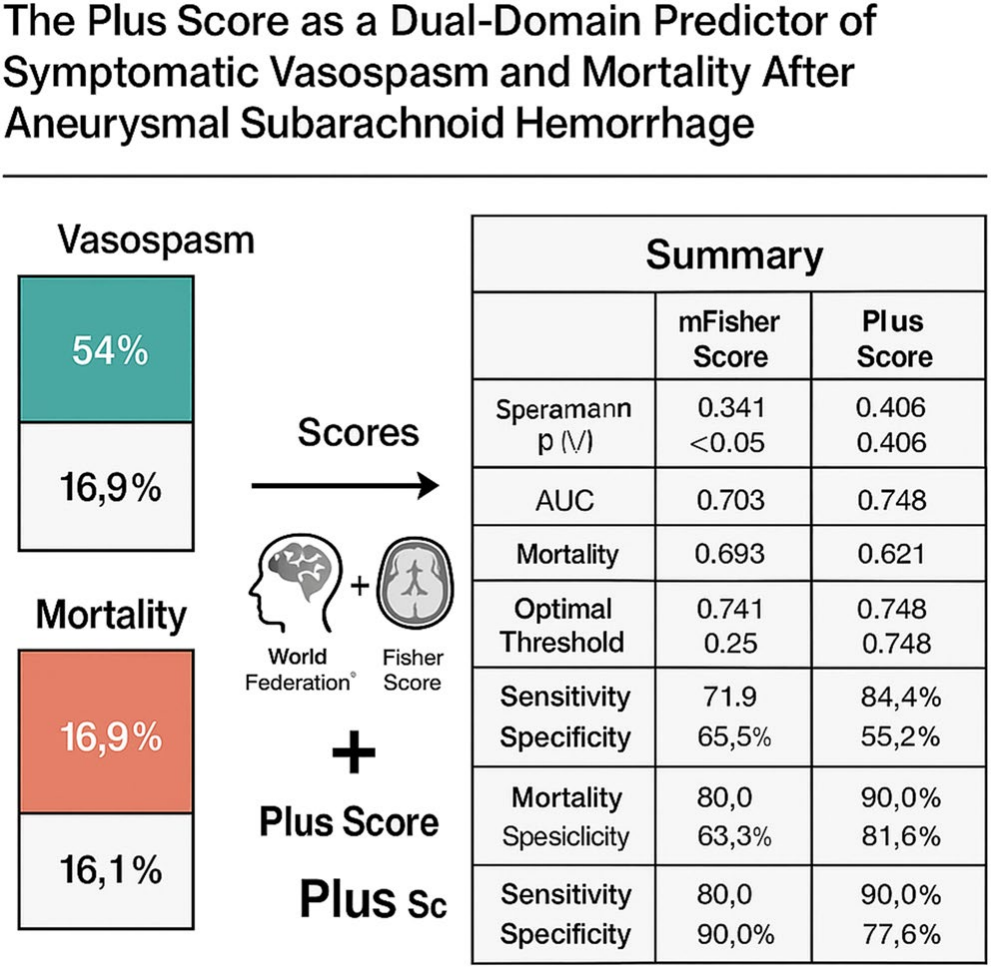

## Introduction

Aneurysmal subarachnoid hemorrhage (aSAH) is a severe form of stroke associated with high early mortality (20–35%) and long-term disability despite advances in treatment [1, 2]. Survivors of the initial hemorrhage remain at risk for secondary complications, particularly cerebral vasospasm leading to delayed cerebral ischemia (DCI), which occurs in approximately 30% of cases between days 3–14 post-ictus and significantly worsens outcomes [3, 4]. Early identification of high-risk patients is thus critical for targeted monitoring and intervention to prevent secondary injury.

The patient’s initial neurological status and the amount of subarachnoid blood on the admission computed tomography (CT) scan are consistently identified as the strongest predictors of DCI [3]. In practice, these factors are commonly quantified using the World Federation of Neurosurgical Societies (WFNS) grading scale (a clinical scale based on Glasgow Coma Scale and focal deficits) and the modified Fisher scale (mFisher: a radiographic grading of subarachnoid hemorrhage thickness and intraventricular blood). To improve early risk stratification, composite scoring systems that integrate clinical and radiological severity have been proposed [1]. Among these, the VASOGRADE classification represents the most widely adopted approach, categorizing patients into Green, Yellow, and Red zones based on WFNS and mFisher scores. While VASOGRADE simplifies bedside triage, its categorical structure inevitably compresses heterogeneous clinical–radiological profiles into broad groups, potentially limiting discriminatory performance. A continuous numerical scoring may better capture incremental increases in hemorrhage volume and neurological impairment, thereby offering a more refined assessment of risk.

In this context, we sought to evaluate whether a simple, continuous metric—the “Plus score,” defined as the arithmetic sum of WFNS and modified Fisher scores—could enhance predictive accuracy for both DCI and mortality following aneurysmal subarachnoid hemorrhage. By integrating two independent dimensions of SAH severity into a single value without categorical thresholding, the Plus score may provide a more granular and quantitatively sensitive prognostic tool compared with either component score alone or existing composite models.

## Materials and methods

### Study design and patient population

This retrospective cohort study was conducted at a single tertiary care center between 2021 and 2025. Patients diagnosed with aSAH who underwent surgical clipping and had a minimum of six months of clinical follow-up were included. All patients received standard subarachnoid hemorrhage management, including early surgical clipping and prophylactic oral nimodipine administration at a dose of 60 mg every 4 h for 21 days, unless contraindicated. Early surgical clipping was defined as surgery performed within 72 h of symptom onset. The primary aim of the study was to evaluate the predictive value of the “Plus score,” defined as the sum of the mFisher and the WFNS grade, for the development of DCI. All eligible cases were included consecutively, and no a priori sample size calculation was performed due to the retrospective design. Nevertheless, the achieved sample size was deemed adequate for exploratory statistical modeling based on observed effect sizes. At our institution (a specialized center for neurovascular surgery), ruptured aneurysms are preferentially treated with microsurgical clipping when lesion morphology, location, and the overall clinical status are favorable. Patients treated primarily with endovascular techniques and those with large intraparenchymal hematomas were excluded in order to maintain a homogeneous surgically managed cohort and to minimize treatment-modality–related confounding.

## Inclusion and exclusion criteria

Inclusion criteria were as follows:

- Diagnosis of aneurysmal subarachnoid hemorrhage confirmed by clinical and radiological findings.
- Treatment with microsurgical clipping.
- Availability of at least six months of follow-up data.

Exclusion criteria included:

- Unruptured aneurysms.
- Associated intraparenchymal hematomas.
- Aneurysms treated with endovascular methods (e.g., coiling).
- Incomplete or missing follow-up data.

After applying these criteria, eligible patients were identified and included in the final analysis.

### Definition of DCI and data collection

Patients were divided into two groups based on the presence or absence of DCI [5]. DCI was defined clinically as the occurrence of new or worsening headache, neurological deficit, or altered consciousness during hospitalization that could not be explained by other causes such as rebleeding, hydrocephalus, infection, seizure, or metabolic disturbances (post-SAH day 3 and 15). Although radiologic confirmation was not mandatory for diagnosis, transcranial Doppler ultrasonography or CT angiography was performed in selected cases at the clinician’s discretion. Demographic, clinical, and radiological data were retrospectively extracted from medical records and imaging archives. The mFisher was graded 0–4 based on the amount and distribution of subarachnoid and intraventricular blood on initial CT scans [6]. The WFNS grade was determined based on the Glasgow Coma Scale and the presence of focal neurological deficits [7]. The Plus score was calculated by summing the mFisher score and the WFNS grade for each patient. A different combination of these two scores has been proposed in a prior study (e.g., VASOGRADE) as a reliable predictor of delayed cerebral ischemia [1]. In addition, the VASOGRADE classification was calculated for each patient according to the method described by de Oliveira Manoel et al., in which patients are stratified into Green (WFNS 1–2 and mFisher 1–2), Yellow (WFNS 1–3 and mFisher 3–4), and Red (WFNS 4–5 regardless of mFisher).

Additional variables collected included:

- **Demographics**: Age, sex, and relevant medical history.
- **Hemorrhage severity**: mFisher and WFNS scores.
- **Aneurysm characteristics**: Location (anterior vs. posterior circulation), size (width, height, and calculated area in mm²).
- **Treatment details**: Timing of surgery, perioperative management, including standardized ICU protocols and nimodipine prophylaxis.
- **Clinical outcomes**: Development of symptomatic DCI (primary outcome), postoperative complications, and mortality.

### Statistical analysis

Statistical analyses were conducted using IBM SPSS Statistics (IBM Corp., Armonk, NY, USA). The normality of continuous variables was assessed using the Shapiro–Wilk test. Normally distributed variables were presented as mean ± standard deviation (SD) and compared using the Student’s t-test. Non-normally distributed variables were expressed as median (interquartile range, IQR) and analyzed using the Mann–Whitney U test. Categorical variables were reported as frequencies and percentages and compared using the Chi-square test or Fisher’s exact test, as appropriate.

Receiver operating characteristic (ROC) analysis and correlation coefficients were used to compare the predictive performance of the WFNS grade, modified Fisher grade, and Plus score. Odds ratios (ORs) and 95% confidence intervals (CIs) were reported. The predictive performance of the mFisher, WFNS, and Plus scores was further evaluated using receiver operating characteristic (ROC) curve analysis. The area under the curve (AUC), optimal cut-off values (using Youden’s index), and corresponding sensitivity and specificity values were calculated. A p-value < 0.05 was considered statistically significant. VASOGRADE was analyzed as an ordinal variable (1–3) and included in ROC analyses for DCI and mortality.

### Ethical considerations

This study was approved by the Institutional Review Board of Istanbul Medipol University (Approval No: 1180, Date: 28.11.2024). The study was conducted in accordance with the ethical standards of the Declaration of Helsinki. Given the retrospective nature of the study and anonymization of patient data, the requirement for informed consent was waived.

## Results

**Fig. 1:**
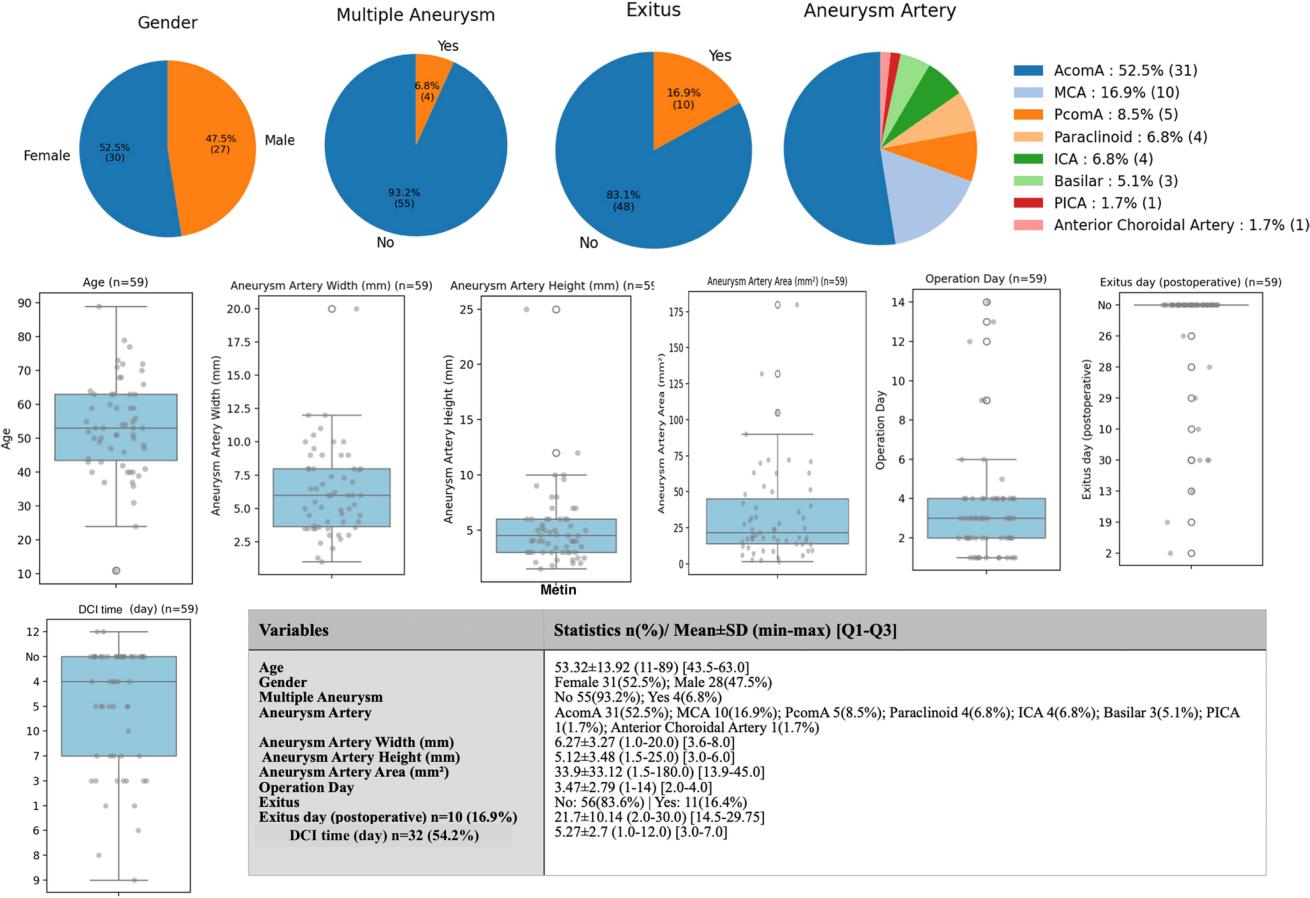
Demographic, clinical, radiological, and perioperative characteristics of patients admitted with aneurysmal subarachnoid hemorrhage. The pie charts in the top row illustrate gender distribution, presence of multiple aneurysms, postoperative mortality, and aneurysm location. The middle section of the figure displays box-plots for continuous variables including patient age, aneurysm width, height, area, and time to surgery. In the lower left corner, DCI onset timing is shown. Finally, the embedded summary table lists key descriptive statistics in the format of n (%), mean ± SD, range, and interquartile range

A total of 59 patients were included in the study, with a mean age of 53.32 ± 13.92 years (range: 11–89) and an interquartile range (IQR) of 43.5–63.0 years. The cohort consisted of 31 female patients (52.5%) and 28 male patients (47.5%). Multiple aneurysms were observed in 4 patients (6.8%), whereas 55 patients (93.2%) had a single aneurysm. The most common aneurysm location was the anterior communicating artery (AcomA) in 31 patients (52.5%), followed by the middle cerebral artery (MCA) in 10 patients (16.9%), and the posterior communicating artery (PcomA) in 5 patients (8.5%). Other aneurysm sites included the paraclinoid region (6.8%), internal carotid artery (ICA, 6.8%), basilar artery (5.1%), posterior inferior cerebellar artery (PICA, 1.7%), and the anterior choroidal artery (1.7%). The mean aneurysm width was 6.27 ± 3.27 mm (range: 1.0–20.0; IQR: 3.6–8.0 mm), and the mean height was 5.12 ± 3.48 mm (range: 1.5–25.0; IQR: 3.0–6.0 mm). The aneurysm area averaged 33.9 ± 33.12 mm², with values ranging from 1.5 to 180.0 mm² (IQR: 13.9–45.0). The mean time from subarachnoid hemorrhage to surgical intervention was 3.47 ± 2.79 days (range: 1–14; IQR: 2.0–4.0). Postoperative mortality (exitus) occurred in 11 patients (16.4%), while 56 patients (83.6%) survived. Among the deceased, the mean postoperative survival time was 21.7 ± 10.14 days (range: 2.0–30.0; IQR: 14.5–29.75). DCI was identified in 32 patients (54.2%) with a mean onset of 5.27 ± 2.7 days postoperatively (range: 1.0–12.0; IQR: 3.0–7.0) (Fig. 1).

Among the 59 patients, DCI was observed in 32 individuals (54.2%), while 27 patients (45.8%) did not develop DCI. Comparative analysis between the two groups revealed that the mFisher score was significantly higher in the DCI group (3.19 ± 0.82 [3.0–4.0]) compared to the non-DCI group (2.37 ± 1.11 [1.0–3.0]; *p* = 0.004). Similarly, the WFNS score was elevated in patients with DCI (2.75 ± 1.74 [1.0–4.0]) compared to those without DCI (1.59 ± 1.28 [1.0–1.0]; *p* = 0.006). The plus score also demonstrated a significant difference, being higher in the DCI group (5.94 ± 2.14 [4.0–8.0]) than in the non-DCI group (4.07 ± 2.07 [2.0–4.0]; *p* = 0.001).

**Fig. 2:**
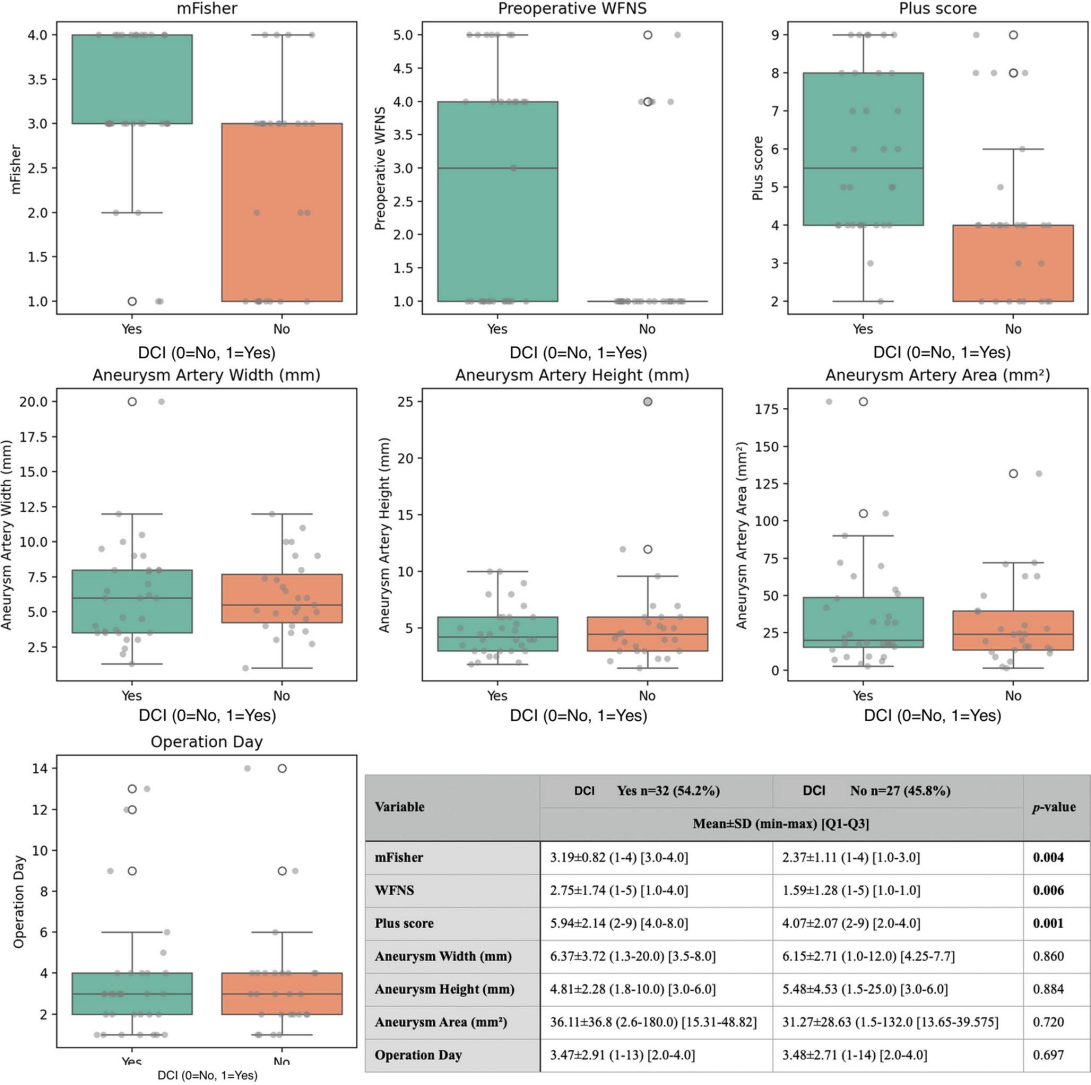
Comparison of clinical and aneurysm-related parameters between patients with and without DCI after aneurysmal subarachnoid hemorrhage. Box-plots illustrate the distribution of preoperative mFisher score, WFNS grade, and the combined Plus score among patients who developed DCI versus those who did not. The table below the plots provides the mean ± standard deviation, minimum–maximum range, and interquartile range [Q1–Q3] for each parameter across both groups, along with associated *p*-values

No statistically significant differences were observed between the groups regarding aneurysm morphometric parameters. Aneurysm width was 6.37 ± 3.72 mm [3.5–8.0] in the DCI group and 6.15 ± 2.71 mm [4.25–7.7] in the non-DCI group (*p* = 0.860). Aneurysm height was 4.81 ± 2.28 mm [3.0–6.0] versus 5.48 ± 4.53 mm [3.0–6.0] (*p* = 0.884), and aneurysm area was 36.11 ± 36.8 mm² [15.31–48.83] versus 31.27 ± 28.63 mm² [13.65–39.58] in the respective groups (*p* = 0.720). Additionally, the timing of surgery did not differ significantly between the groups, with a mean operation day of 3.47 ± 2.91 [2.0–4.0] in the DCI group and 3.48 ± 2.71 [2.0–4.0] in the non-DCI group (*p* = 0.697) (Fig. 2).

Of the 59 patients, 10 (16.9%) died during the postoperative course (exitus group), while 49 (83.1%) survived. DCI was significantly more common in the exitus group (100%, *n* = 10) compared to the survival group (22 out of 49 patients; 44.9%) (*p* = 0.001). The mean mFisher grade was significantly higher among exitus patients (3.5 ± 0.71 [3.0–4.0]) than survivors (2.67 ± 1.05 [2.0–3.0]; *p* = 0.015). Additionally, WFNS scores were markedly elevated in patients who died (4.3 ± 1.25 [4.0–5.0]) compared to those who survived (1.8 ± 1.37 [1.0–3.0]), although it was statistically significant (*p* = 0.0001).

**Fig. 3:**
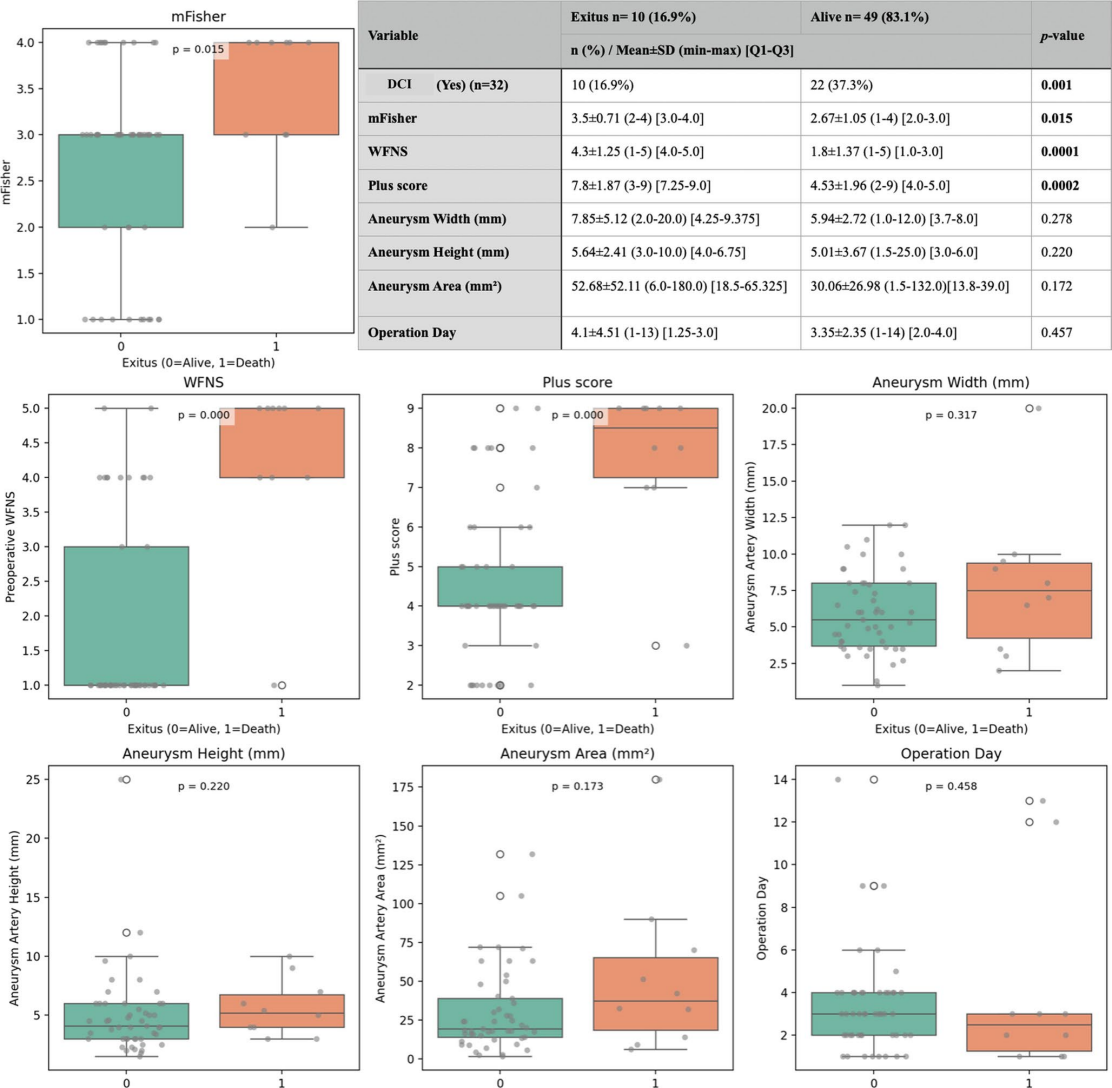
Comparison of clinical, radiological, and aneurysm morphometric parameters between deceased and surviving patients following aneurysmal subarachnoid hemorrhage. Boxplots depict the distribution of modified Fisher scores, WFNS grades, and Plus scores, all of which were significantly higher in the exitus group. The corresponding table provides the detailed summary statistics including frequency, mean ± standard deviation, range, interquartile range, and p-values for each variable

The plus score, reflecting overall clinical burden, was also significantly higher in the exitus group (7.8 ± 1.87 [7.25–9.0]) compared to survivors (4.53 ± 1.96 [4.0–5.0]; *p* = 0.0002). Morphometric parameters of aneurysms, including aneurysm width (7.85 ± 5.12 mm vs. 5.94 ± 2.72 mm; *p* = 0.278), height (5.64 ± 2.41 mm vs. 5.01 ± 3.67 mm; *p* = 0.220), and area (52.68 ± 52.11 mm² vs. 30.06 ± 26.98 mm²; *p* = 0.172), were all higher in the exitus group but did not reach statistical significance. Similarly, the time to surgery was slightly longer in patients who died (4.1 ± 4.51 days) compared to survivors (3.35 ± 2.35 days; *p* = 0.457), though this difference was not statistically significant (Fig. 3).

**Fig. 4:**
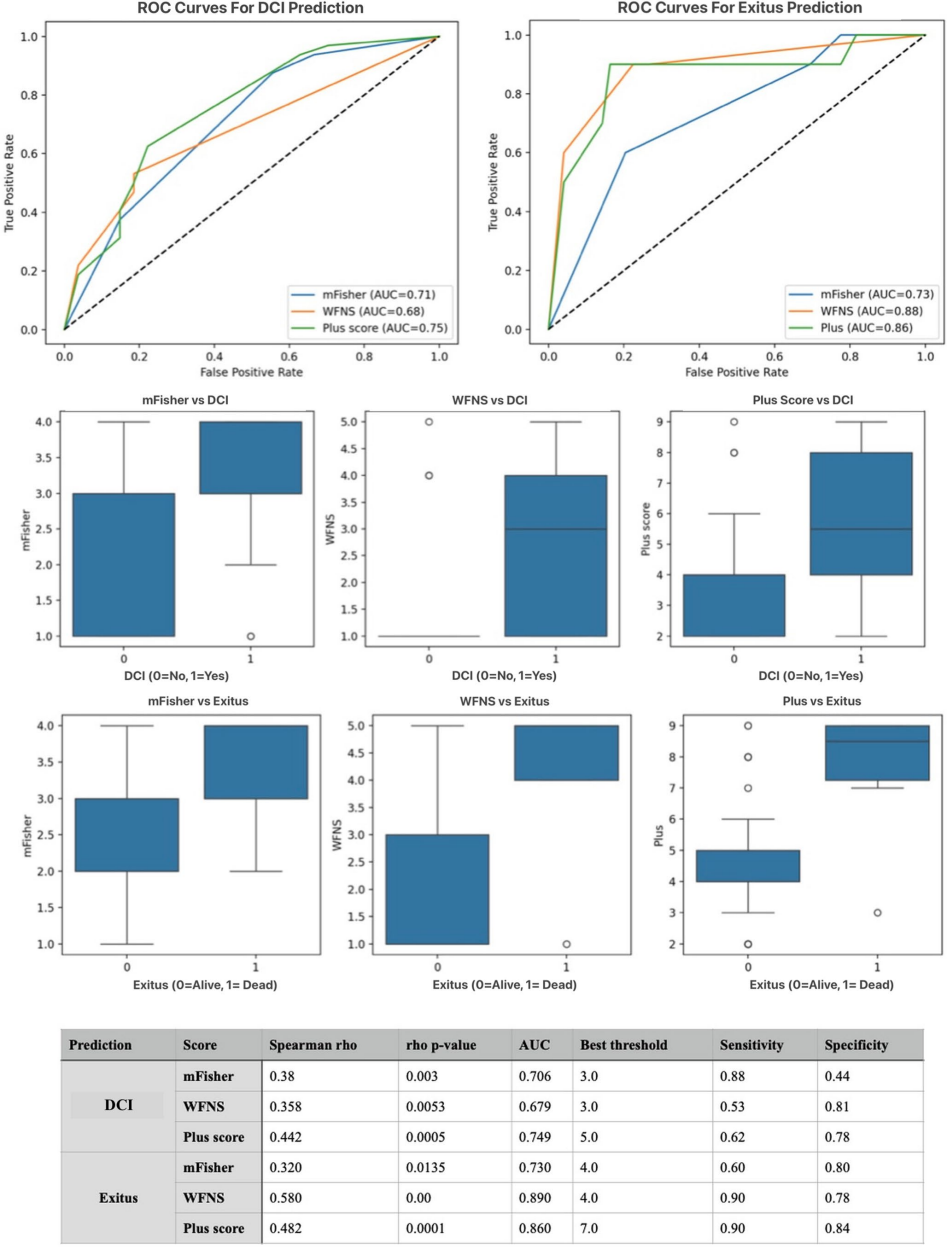
Predictive performance and distribution of mFisher, WFNS, and Plus scores for DCI and mortality in aneurysmal subarachnoid hemorrhage. Predictive performance of mFisher score, WFNS grade, and the combined Plus score for DCI and in-hospital mortality (exitus) in patients with aneurysmal subarachnoid hemorrhage (SAH). The accompanying table summarizes Spearman correlation coefficients (rho), p-values, AUC values, optimal thresholds (determined via Youden’s index), and corresponding sensitivity and specificity for each score in predicting DCI and exitus

The predictive value of mFisher, WFNS, and Plus scores for DCI and mortality (exitus) was assessed using Spearman correlation and ROC curve analysis. The Plus score demonstrated the strongest correlation with DCI (*r* = 0.406) and mortality (*r* = 0.621), followed by WFNS (*r* = 0.351 and *r* = 0.543, respectively) and mFisher score (*r* = 0.341 and *r* = 0.393, respectively). Receiver operating characteristic (ROC) analysis demonstrated that the Plus score had the strongest discriminative performance for predicting both DCI and mortality. For DCI prediction, the Plus score yielded an AUC of **0.748 (95% CI: 0.63–0.86)** with an optimal cut-off of 4.5 (sensitivity 84.4%, specificity 55.2%). WFNS showed an AUC of **0.704 (95% CI: 0.58–0.82)**, and the modified Fisher scale had an AUC of **0.703 (95% CI: 0.57–0.82)**. For mortality prediction, the Plus score again outperformed the individual scales, with an AUC of **0.870 (95% CI: 0.77–0.96)** and a cut-off of 5.5 (sensitivity 90.0%, specificity 77.6%). WFNS demonstrated an AUC of **0.858 (95% CI: 0.75–0.94)**, while the modified Fisher grade showed a lower discriminative capacity with an AUC of **0.741 (95% CI: 0.59–0.87)**. When compared with VASOGRADE, the Plus score consistently demonstrated superior discriminative accuracy for both outcomes. VASOGRADE showed an AUC of **0.691 (95% CI: 0.55–0.82)** for DCI and **0.734 (95% CI: 0.61–0.87)** for mortality, both lower than the corresponding AUCs of the continuous Plus score (Fig. 4).

**Fig. 5:**
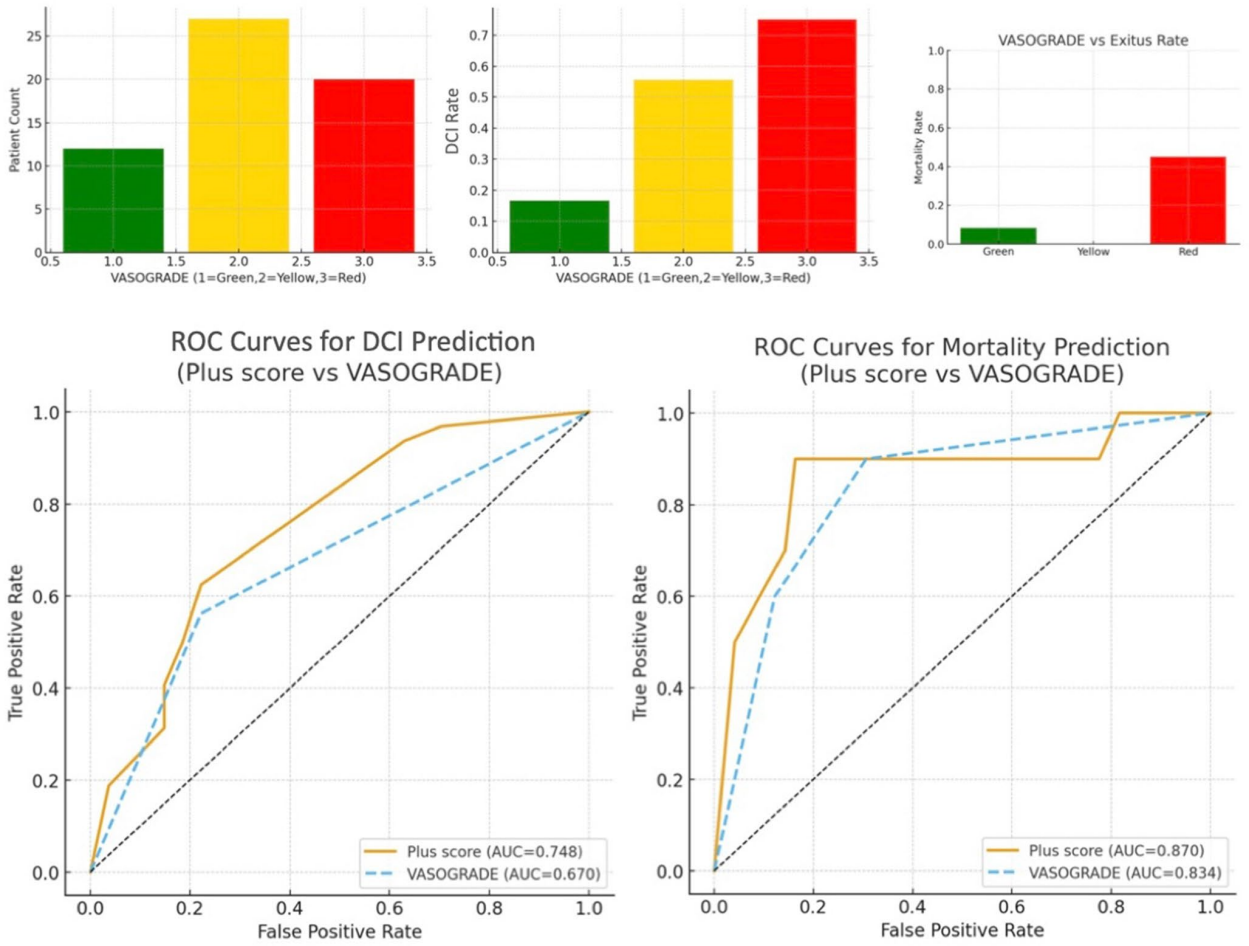
Comparison of Plus score and VASOGRADE for predicting DCI and mortality. The distribution of mortality across VASOGRADE categories (Green, Yellow, Red) further showed progressively increasing mortality rates toward the Red category. Overall, these findings indicate that the continuous Plus score provides a more sensitive and granular assessment of clinical and radiological severity than the categorical VASOGRADE system. The ROC analysis demonstrated that the Plus score exhibited superior discriminative performance for DCI prediction compared with VASOGRADE. Similarly, for mortality prediction, the Plus score again outperformed VASOGRADE, yielding the highest AUC value

In the comparative ROC analysis evaluating the predictive performance of the Plus score versus VASOGRADE for both DCI and mortality, the Plus score demonstrated superior discriminative ability across outcomes. For DCI prediction, the Plus score achieved an AUC of 0.748 with an optimal cut-off of 4.5, outperforming VASOGRADE, which yielded a lower AUC. Although 4.5 was identified as the statistically optimal threshold, using a rounded cut-off of 5 may be more practical in real-world settings. Such a cut-off still correctly captures the majority of high-risk patients while enhancing clinical usability. Similarly, for mortality prediction, the Plus score reached an AUC of 0.870—again higher than that of VASOGRADE—highlighting its stronger prognostic accuracy. These findings indicate that while VASOGRADE provides a categorical stratification of clinical–radiological severity, the continuous structure of the Plus score offers a more sensitive and granular assessment, resulting in enhanced predictive performance for both DCI and postoperative fatal outcome (Fig. 5).

## Discussion

This study assessed the predictive value of the “Plus score”—the arithmetic sum of the WFNS and modified Fisher grades—for DCI and mortality following aneurysmal SAH treated with microsurgical clipping. Our results showed that the Plus score was strongly associated with DCI and moderately associated with mortality. These findings align with existing evidence that both neurological status and hemorrhage burden are key determinants of delayed DCI and outcomes after SAH [8, 9].

The individual predictive power of WFNS and mFisher scores has been widely validated. Poor neurological grade at admission, measured by WFNS or Hunt–Hess, correlates with higher DCI incidence and worse outcomes [10, 11]. Likewise, subarachnoid blood volume, particularly when accompanied by intraventricular hemorrhage (IVH), elevates DCI risk [6, 12]. However, the original Fisher scale grouped thick cisternal blood and IVH into a single category, limiting its discriminatory capacity. Frontera et al. demonstrated that the modified Fisher scale better predicts DCI, with each grade associated with increased odds [6]. Our study supports these findings, as both individual components of the Plus score were higher in patients who developed DCI.

Furthermore, our findings are in line with the broader literature supporting dual-domain scoring systems. The VASOGRADE model, introduced by de Oliveira Manoel et al., stratifies patients by WFNS and modified Fisher into Green, Yellow, and Red zones and was validated in a 746-patient cohort, with Red-zone patients having a threefold increased risk of DCI [1]. Although VASOGRADE provides categorical stratification, our Plus score presents a continuous measure, enhancing granularity while preserving simplicity. Similarly, Chen et al. found that only combined scores like VASOGRADE retained independent significance in predicting DCI, unlike WFNS or modified Fisher alone [13]. Despite the use of VASOGRADE for early risk stratification, our findings suggest that its categorical structure may limit its predictive resolution, particularly in borderline clinical–radiological profiles. The Plus score, by functioning as a continuous dual-domain metric, provides a more nuanced reflection of combined neurological and hemorrhagic burden. In our cohort, the Plus score consistently outperformed VASOGRADE in ROC analysis for both DCI and mortality, indicating that finer gradations captured by continuous scoring may translate into improved prognostic accuracy. Importantly, while VASOGRADE remains a practical bedside tool, our results imply that it may underestimate risk in patients whose WFNS and modified Fisher values lie close to category thresholds. This supports previous observations that composite indices with broader numerical ranges better accommodate the heterogeneity of early SAH presentations. Future prospective studies should examine whether integrating the Plus score into existing triage frameworks can enhance risk-adapted monitoring and early intervention strategies.

Our study also showed that the Plus score had moderate predictive power for mortality. This is biologically plausible, as patients with high WFNS and dense SAH (i.e., high Plus score) likely sustain more severe injury. The correlation between high WFNS grade and mortality is well established [14]. Our data also suggest that the association between the Plus score and mortality may be mediated through DCI: all patients who died experienced DCI, whereas less than half of the survivors did. However, the moderate discrimination suggests that outcome is multifactorial—some low-risk patients may die from unrelated complications, and not all high-risk patients succumb. This is consistent with literature emphasizing the role of factors like age, comorbidities, and systemic complications in mortality risk [15].

Recent risk models further validate our approach. Chen et al. developed a nomogram using WFNS, modified Fisher, intraventricular blood, and early brain edema as predictors, achieving an AUC of 0.86 [16]. This aligns with our findings, where the Plus score—based on two of those core predictors—showed comparable accuracy. Likewise, a 2024 machine-learning study by Azzam et al. identified clinical DCI as the most important predictor of DCI, confirming the central role of hemorrhage burden and neurological status [4]. While advanced models may offer marginal improvements, their complexity limits bedside applicability. In contrast, the Plus score is easy to calculate and interpret, offering transparent and actionable risk assessment.

Radiologic studies echo these findings. A European Radiology (2024) analysis of eight CT-based SAH grading scales found all but the original Fisher scale correlated with DCI [9]. They observed that poor-grade patients (WFNS 3–5) had higher DCI rates and that large clot burden amplified risk even in good-grade patients. This supports our observation that DCI risk arises from both clinical and radiographic severity, and the Plus score captures this interaction efficiently. It serves as a proxy for overall SAH severity, with practical advantages over more complex scoring systems. As de Oliveira Manoel et al. noted, prognostic models requiring many variables often offer only marginal gains, hindering their adoption [1]. In contrast, the Plus score uses familiar tools—WFNS and modified Fisher—and communicates risk clearly (e.g., “Plus score 8” implies WFNS 4 + Fisher 4).

Clinically, the Plus score has clear utility. Patients with high scores may benefit from closer ICU monitoring, frequent neurochecks, early transcranial Doppler (TCD) screening, or even preemptive angiography [17]. A high Plus score can trigger early interventions, while low scores may prompt consideration of alternate diagnoses if deterioration occurs. Moreover, in high-Plus-score patients, clinicians may initiate discussions about anticipated clinical course and escalate therapy promptly when needed. Given its high sensitivity but modest specificity (55.2%), the Plus score may be more appropriate as a clinical screening tool to identify patients at elevated risk for DCI rather than a definitive diagnostic metric. Patients flagged by the Plus score would still require confirmatory imaging and clinical evaluation.

This study has several limitations that should be acknowledged. First, its retrospective, single-center design introduces inherent risks of selection bias and limits the generalizability of the findings. The overall sample size was modest, and the number of mortality events was particularly small (*n* = 10), which reduces the precision and stability of mortality-related effect estimates, including AUC values. The limited number of deaths precluded robust multivariable analysis for mortality. Second, the cohort was restricted to patients who underwent microsurgical clipping within 72 h of ictus; therefore, the results may not be applicable to patients treated with endovascular coiling or flow-diverting techniques, or to those managed outside the early intervention window. Third, DCI was defined clinically without mandatory radiographic confirmation, and systematic use of CTA, DSA, or perfusion imaging was not performed. This could have resulted in misclassification, particularly in cases with sedation, fluctuating exams, or microcirculatory dysfunction. Fourth, although we evaluated the Plus score against established grading systems, the analysis included limited adjustment for other prognostic variables such as age, comorbidities, aneurysm morphology, or variations in perioperative management. Because DCI was defined clinically, neurological deterioration may have been confounded by other causes such as seizures, hydrocephalus, metabolic disturbances, or sedation, potentially leading to false-positive classification. Lastly, no external or temporal validation was performed; hence, the predictive performance of the Plus score should be interpreted as exploratory and requires confirmation in larger, prospectively collected, multicenter cohorts.

## Conclusion

The Plus score was associated with the occurrence of delayed cerebral ischemia and in-hospital mortality in patients with aneurysmal SAH and may provide improved early risk stratification compared with the WFNS grade or modified Fisher scale alone. However, these findings should be interpreted cautiously given the retrospective single-center design, limited sample size, and lack of external validation. The Plus score should be considered a simple and accessible bedside aid rather than a replacement for comprehensive clinical judgment or more sophisticated prognostic models. Prospective multicenter studies are needed to confirm its predictive utility and to determine how it may best be integrated into existing risk stratification frameworks. 

## Data Availability

The datasets generated and/or analyzed during the current study are available from the corresponding author upon reasonable request.

## Abbreviations

WFNS: World Federation of Neurosurgical Societies
SAH: Subarachnoid Hemorrhage
DCI: Delayed Cerebral Ischemia
CT: Computed Tomography
IVH: Intraventricular Hemorrhage
ICU: Intensive Care Unit
AUC: Area Under the Curve
OR: Odds Ratio
MRI: Magnetic Resonance Imaging
SAHIT: Subarachnoid Hemorrhage International Trialists
VASOGRADE: Vasospasm Grading System combining WFNS and modified Fisher
TCD: Transcranial Doppler
CI: Confidence Interval
